# Antiviral and Immunomodulatory Effects of α-Mangostin Against Feline Infectious Peritonitis Virus: In Vitro Assay

**DOI:** 10.3390/ani15162417

**Published:** 2025-08-18

**Authors:** Varanya Lueangaramkul, Pratipa Termthongthot, Natjira Mana, Pharkphoom Panichayupakaranant, Ploypailin Semkum, Porntippa Lekcharoensuk, Sirin Theerawatanasirikul

**Affiliations:** 1 Graduate Program in Animal Health and Biomedical Sciences, Faculty of Veterinary Medicine, Kasetsart University, Bangkok 10900, Thailand; varanya.luea@ku.th (V.L.); pratipa.te@ku.th (P.T.); 2 Department of Anatomy, Faculty of Veterinary Medicine, Kasetsart University, Bangkok 10900, Thailand; 3 Department of Microbiology and Immunology, Faculty of Veterinary Medicine, Kasetsart University, Bangkok 10900, Thailand; natjira.ma@ku.th (N.M.); fvetpls@ku.ac.th (P.S.); fvetptn@ku.ac.th (P.L.); 4 Department of Pharmacognosy and Pharmaceutical Botany, Faculty of Pharmaceutical Sciences, Prince of Songkla University, Hat-Yai, Songkhla 90112, Thailand; pharkphoom.p@psu.ac.th; 5 Phytomedicine and Pharmaceutical Biotechnology Research Center, Faculty of Pharmaceutical Sciences, Prince of Songkla University, Hat-Yai, Songkhla 90112, Thailand

**Keywords:** Feline infectious peritonitis virus, α-mangostin, bioactive compounds, antiviral activity, in vitro screening, cytokine modulation, drug combination

## Abstract

Feline infectious peritonitis virus (FIPV), caused by a mutated form of feline coronavirus, poses a significant threat to feline health worldwide, with limited therapeutic options available. This study investigated the antiviral potential of α-mangostin (α-MG) and its enriched extracts (AMEs), obtained via microwave-assisted extraction, against FIPV. We evaluated their cytotoxicity, direct virucidal activity, and antiviral activity in CRFK cells. Both α-MG and AMEs demonstrated significant antiviral activity, with EC_50_ values from 2.71 to 2.88 μg/mL and favorable selectivity indices (3.25–3.66). Notably, AMEs exhibited direct virucidal effects, effectively reducing viral titers. Furthermore, treatment with these compounds significantly reduced inflammatory cytokine expression (IFN-β, TNF-α, and IL-6 mRNA levels) and decreased viral loads in FIPV-infected cells. Drug combination studies using the ZIP model revealed enhanced cooperative effects when AMEs and α-MG were combined with GC-376 or GS-441524, with GC-376 combinations showing particularly strong synergistic potential. These findings suggest that α-MG and AMEs are promising candidates for FIPV treatment, either as monotherapy or in combination therapy. This study provides insights into developing novel therapeutic strategies to combat FIPV infections and offers a foundation for future veterinary antiviral drug development.

## Simple Summary

Feline infectious peritonitis (FIP) is a deadly disease in cats, and currently available antiviral drugs are illegal, expensive, and almost inaccessible. Our research explored a new, safer solution using natural compounds called α-mangostin, found in the thick skin of mangosteen fruit. We used eco-friendly methods to extract these compounds and tested their ability to combat the FIP virus and reduce viral-induced, harmful inflammation. Our findings showed that α-mangostin and its extracts effectively stopped the virus multiplication while alleviating inflammation mediators. Importantly, these natural compounds also worked well when combined with existing antiviral drugs, potentially making treatments even more effective. This discovery provides a promising new path for veterinarians and cat owners, paving the way for safer and more effective therapies for cats affected by this devastating illness.

## 1. Introduction

Feline coronavirus (FCoV) is an enveloped, single-stranded, positive-sense RNA virus belonging to the *Alphacoronavirus* genus, representing a significant challenge in veterinary medicine [1,2]. Two distinct FCoV biotypes have been identified: avirulent feline enteric coronavirus (FECV) and virulent feline infectious peritonitis virus (FIPV). While FECV primarily replicates within enterocytes and causes mild enteric signs, FIPV represents a more severe manifestation, replicating within monocytes and tissue macrophages and leading to systemic spread [3,4]. In the systemic response to FIP, infected cats show increased levels of inflammatory cytokines and chemokines across multiple affected organs [5].

Feline infectious peritonitis (FIP) is a progressive disease with a historically poor prognosis. This necessitates the development of effective therapeutic interventions [2]. While recent progress in antiviral drug development, such as the protease inhibitor GC-376 and the nucleoside analog GS-441524, has shown promising clinical efficacy in treating FIP cases [6,7,8,9], it is crucial to note that these agents remain unapproved, unlicensed, and often inaccessible in many regions [2]. This leads to issues with inconsistent product quality, high costs, and variations in clinical outcomes, including concerns about relapse [2,6,7,8,9]. Furthermore, the intrinsic characteristics of FCoV, including its high mutation rate and capacity to generate genetically diverse populations through mutation and recombination within individual cats, continue to present challenges for therapeutic interventions. The virus’s capacity to mutate from the enteric to systemic form within individual cats emphasized the complexity of developing targeted treatment strategies, highlighting the urgent need for accessible, safe, and consistently effective treatment options [1,2].

The exploration of natural compounds as antiviral agents has gained attention in veterinary therapeutics due to their potential for novel mechanisms of action and reduced toxicity. Our previous research demonstrated the antiviral potential of bioactive compounds from flavonoids against FIPV [10]. *Garcinia mangostana* L. (mangosteen), a tropical fruit tree indigenous to Southeast Asia, represents a rich source of bioactive xanthones [11]. The fruit’s pericarp contains high concentrations of prenylated xanthones, with α-mangostin (α-MG) being the predominant compound [12]. This xanthone exhibits diverse pharmacological activities, including antioxidant, anti-inflammatory, and antimicrobial properties [12,13,14].

α-MG has demonstrated broad-spectrum antiviral activity against various human viral pathogens, including human immunodeficiency virus (HIV) [15], rotavirus [16], hepatitis C virus (HCV) [17], dengue virus (DENV) [18], chikungunya virus (CHIKV) [19], and SARS-CoV-2 [20]. The compound’s anti-inflammatory properties suggest additional therapeutic value through modulation of host immune responses during viral infections [21], which is particularly relevant in FIP pathogenesis, where excessive inflammatory responses contribute to disease severity. Despite these promising findings, research investigating mangosteen-derived compounds against veterinary viral pathogens remains limited.

The present study aims to evaluate the antiviral and immunomodulatory effects of both pure α-MG and its α-mangostin-rich extracts (AMEs) obtained through green extraction technology against feline infectious peritonitis virus. The evaluation of AMEs is particularly significant due to their production via eco-friendly methods, potentially offering a safer profile, and the possibility of enhanced therapeutic efficacy from cooperative actions among their phytoconstituents. The therapeutic potential is explored using either the compound alone or in combination with currently recognized antiviral agents, providing insights into potential therapeutic applications for this challenging feline disease.

## 2. Materials and Methods

### 2.1. Plant Materials and Compound Preparation

*Garcinia mangostana* fruits were processed following established protocols [22,23]. Briefly, fruit pericarps were cut into small pieces, dried at 60 °C for 72 h, pulverized, and sieved through a No. 20 sieve to obtain fine powder. Green extraction was performed using polyethylene glycol (PEG) 400 under optimized microwave conditions (2450 MHz, 900 watts, 24 min, 90 °C). The mixture was filtered and fractionated using a Diaion HP-20 column with sequential elution using 60% and 70% (*v*/*v*) ethanol. Fractions from 70% ethanol elution were dried using a rotary evaporator at 45 °C to obtain a yellow powder of α-mangostin-rich extracts (AMEs).

α-MG content was quantified using reversed-phase HPLC with a C18-pentafluorophenyl column and mobile phase of acetonitrile: 0.2% formic acid (70:30, *v*/*v*). Calibration curves were constructed using authentic α-MG (12.5–200 μg/mL). Two AMEs were obtained: AME95 (95.6% *w*/*w* α-MG) and AME84 (83.6% *w*/*w* α-MG) [14,23]. Purified α-MG (>98% purity) served as a reference compound [22]. GC-376 and GS-441524 (TargetMol, Boston, MA, USA) were used as positive controls for antiviral assays and drug combination studies.

### 2.2. Cell Culture and Virus Preparation

CRFK cells (ATCC^®^, Manassas, VA, USA) were cultivated in Minimum Essential Medium supplemented with 10% fetal bovine serum (FBS, MEM, Gibco^™^, Thermo Fisher Scientific Inc., Waltham, MA, USA) and maintained at 37 °C in a humidified atmosphere containing 5% CO_2_. FIPV strain WSU 79-1146 (ATCC^®^, Manassas, VA, USA) was propagated in the CRFK cells as previously described [24]. Cytopathic effect was characterized by multinucleated giant and rounded cells observed under an inverted microscope (Olympus IX73, Tokyo, Japan) as described previously [10]. Viral titer was determined using the Reed and Muench method [25] to calculate the 50% tissue culture infectious dose (TCID_50_) per mL. The virus stock with a titer of 1 × 10^7^ TCID_50_/mL was aliquoted and stored at –80 °C for subsequent experiments.

### 2.3. Cytotoxicity Assay

Cell viability was evaluated using the CCK-8 assay according to the manufacturer’s instructions (Abbkine Scientific Co.,Ltd., Atlanta, GA, USA). The CRFK cells were seeded at 2.2 × 10^4^ cells/well in 96-well plates containing MEM supplemented with 10% FBS and incubated overnight. The medium was then replaced with serially diluted concentrations (0.01, 0.1, 1, 2.5, 5, and 10 µg/mL) in 2% FBS MEM, and the plates were incubated at 37 °C in 5% CO_2_ for 24 h.

For cell viability evaluation, the medium containing compounds was removed, and cells were washed with 1× rinse saline [24,26]. Fresh MEM was added to each well, followed by 10 µL of CCK-8 solution. The plates were further incubated for 2 h, and optical density (OD) was measured at 450 nm using a multimode reader (Synergy H1 Hybrid Multi-Mode Reader, BioTek^®^, Winooski, VT, USA). Data were blank-subtracted and normalized to untreated cell controls (set as 100%). The half-maximal cytotoxic concentration (CC_50_) was calculated as the concentration required to reduce cell viability by 50%.

(1) $$\frac{[OD\;treaded-OD\;cell\;control]\;}{\left[OD\;dmso-OD\;cell\;control\right]}\times 100$$

### 2.4. Antiviral Activity Assays

Antiviral activity was evaluated at different stages of FIPV infection using modified previously described methods with slight modifications [10,24]. The CRFK cells (2.2 × 10^4^ cells/well) were seeded in 96-well plates and incubated overnight. For protection assays, cells were pre-treated with serially diluted compounds for 2 h before FIPV infection (50 TCID_50_/100 µL/well) for 2 h. In binding assays, compound–virus mixtures were added to cells for 2 h. For post-infection assays, cells were infected with FIPV for 2 h, followed by compound treatment.

After infection, the inoculum was removed, the cells were washed with 1× rinse saline [24,26], fresh medium was added, and the cells were incubated. Antiviral activity was evaluated using immunoperoxidase monolayer assay (IPMA) at 24 h and cytopathic effect (CPE) reduction with 0.5% crystal violet staining at 48 h [10,24]. For direct virucidal activity assessment, the virus was incubated with compounds at 37 °C for 1 h, and then the mixtures were serially diluted 10-fold and added to cells for 3 days. Viral titers were determined using the Reed and Muench method [25].

### 2.5. Immunoperoxidase Monolayer Assay (IPMA)

IPMA was performed according to previous protocols [24,26]. The cells were fixed with cold methanol at room temperature for 30 min and washed with PBS containing 0.05% Tween (PBST). The fixed cells were treated with 50 µL/well blocking buffer (BlockPRO^™^ 1 Min Protein-Free Blocking Buffer, Taipei, Taiwan) for 1 min, and then incubated with primary antibody (mouse anti-FIPV3-70, 1:500 dilution; ThermoFisher, Carlsbad, CA, USA) at 37 °C for 1 h. Following PBST washing, the cells were incubated with goat anti-mouse IgG-HRP antibody (1:500 dilution; Thermo Fisher, Waltham, MA, USA) at 37 °C for 1 h [10,24]. FIPV antigens were visualized using DAB substrate (DAKO, Santa Clara, CA, USA), which developed a brown color in infected cell cytoplasm. Images were captured using a phase-contrast inverted microscope (Olympus IX73, Tokyo, Japan) and analyzed using CellProfiler version 4.2.0 (Broad Institute, Cambridge, MA, USA) to quantify positive signals. Data were normalized as percentages of positive infections relative to controls, with mock-infected cells as 0% infection and FIPV-infected cells as 100% infection. The half-maximal effective concentration (EC_50_) was calculated as the concentration required to reduce viral infection by 50%.

### 2.6. Viral Load Quantification and Cytokine mRNA Expression Analysis by RT-qPCR

The CRFK cells were seeded at 2 × 10^5^ cells/well in 24-well plates overnight, and then infected with FIPV inoculum for 2 h. Viral RNA levels in the compound-treated, FIPV-infected cells were quantified and compared to viral-infected and mock-infected controls using RT-qPCR. Total RNA was extracted using TRIzol reagent (Invitrogen™, Carlsbad, CA, USA) and purified using the Direct-zol RNA MiniPrep kit (Zymo Research Corporation, Tustin, CA, USA). Complementary DNA (cDNA) was synthesized from 1 µg total RNA using RevertAid reverse transcriptase (Thermo Fisher, Waltham, MA, USA) with random hexamers for viral load quantification or oligo-dT primers for cytokine mRNA expression analysis [10,24].

The qPCR reaction mixture contained 5 µL iTaq Universal SYBR Green Supermix (2×) (Bio-Rad Laboratories, Hercules, CA, USA), 1 µL cDNA template, and 0.5 µL each of forward and reverse primers (Table 1). Amplification was performed using a C1000 Touch thermal cycler (Bio-Rad Laboratories, Hercules, CA, USA) with initial denaturation at 95 °C for 30 s, followed by 30 cycles of denaturation at 95 °C for 5 s and annealing/extension at 60 °C for 5 s. A melting curve analysis was conducted from 65 °C to 95 °C with 0.5 °C increments [10,24]. All the analyses were performed using two biological replicates with three technical replicates each. 

Viral copy numbers were determined using a standard curve generated from 10-fold serial dilutions (10^−2^ to 10^−7^ molecules/µL) of a plasmid containing the FIPV 3′UTR sequence. Cytokine mRNA expression levels were quantified using the 2^−ΔΔCT^ method [27], with glyceraldehyde-3-phosphate dehydrogenase (GAPDH) as the internal reference gene.

**Table 1:** Primers used in this study.

Genes	Primer Sequences (5′-3′)	Product Size	Accession Numbers and References
Viral load quantification			
FCoV 3′UTR-F	GGCAACCCGATGTTTAAAACTGG	210	DQ010921.1 [28]
FCoV 3′UTR-R	CACTAGATCCAG ACGTTAGCTC		
mRNA cytokines			
fe-TNF-α-F	TGGCCTGCAACTAATCAACC	251	NM_001009835.1 [29]
fe-TNF-α-R	GTGTGGAAGGACATCCTTGG		
fe-IFN-β-F	GAAGGAGGAAGCCATATTGGT	172	NM_001009297.1 [30]
fe-IFN-β-R	CTCCATGATTTCCTCCAGGAT		
fe-IL-6-F	CCCTGCAGACAAAATGGAAGA	110	L16914.1 [31]
fe-IL-6-R	GTGCCTCCTTGCTGTCCTCA		
fe-GAPDH-F	CATCAATGGAAAGCCCATCAC	97	NM 001009307.1 [32]
fe-GAPDH-R	CCCAGTAGACTCCACAACATAC		

### 2.7. Drug Combination Assay

Combined antiviral effects of α-MG and AMEs (AME95 and AME84) with GS-441524 and GC-376 were evaluated using a 5 × 5 dose–response matrix in FIPV-infected CRFK cells. The cells were seeded in 96-well plates at 2.2 × 10^4^ cells/well and incubated for 24 h at 37 °C with 5% CO_2_. The cells were infected with FIPV (50 TCID_50_/100 µL/well) for 2 h, after which the virus inoculum was removed and fresh medium containing drug combinations was added. Serial dilutions created five concentrations each, with GS-441524 and GC-376 diluted along columns and α-MG and AMEs along rows, creating 25 unique combinations per plate in duplicate. Drug combinations were evaluated using two methods. Cell viability was quantitatively measured using CCK-8 assay after 48 h treatment, with absorbance measured at 450 nm. Subsequently, the plates were fixed with cold methanol and stained with 0.5% crystal violet for qualitative assessment of cell monolayer integrity [10].

A drug interaction analysis was performed using SynergyFinder version 3.0 (https://synergyfinder.fimm.fi, accessed on 16 May 2025). The Zero Interaction Potency (ZIP) model evaluated potential synergistic effects. Synergy scores were calculated based on the difference between observed and expected combination effects, where scores >10 were strongly synergistic, −10 to 10 were moderately synergistic or additive, and <−10 were antagonistic [33].

### 2.8. Statistical Analysis

Statistical analyses were performed using GraphPad Prism version 8.4.1 (GraphPad Software, San Diego, CA, USA). CC_50_ and EC_50_ values were determined using non-linear regression analysis. Data are presented as mean ± standard deviation (SD). Differences between groups were evaluated using one-way ANOVA followed by Tukey’s post hoc test. Statistical significance was set at *p* < 0.05. All the experiments were conducted in duplicate and repeated in at least three independent experiments.

## 3. Results

### 3.1. Cytotoxicity of α-MG and α-Mangostin-Rich Extracts (AMEs)

**Figure 1:**
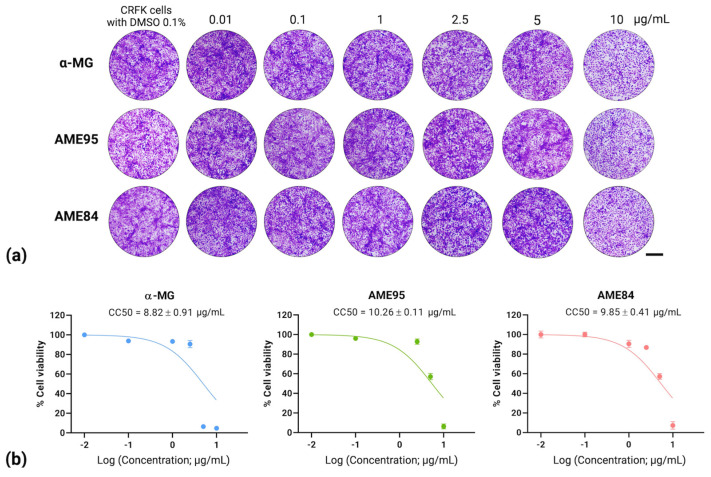
Cytotoxicity of α-MG and AMEs on CRFK cells: (**a**) Cell morphology after 24 h treatment visualized by 0.5% crystal violet staining. Scale bar = 200 µm. (**b**) Dose–response curves showing cell viability and CC_50_ values.

The cytotoxicity of α-MG and AMEs was evaluated using the CCK-8 cell viability assay in CRFK cells. Both AME preparations exhibited lower cytotoxicity compared to the reference compound α-MG (Figure 1). AME95 and AME84 showed CC_50_ values of 10.26 ± 0.11 µg/mL and 9.85 ± 0.41 µg/mL, respectively, while α-MG demonstrated a CC_50_ value of 8.82 ± 0.91 µg/mL (Table 2). The concentration–response curves revealed dose-dependent cytotoxic effects for all compounds, with cell viability remaining above 80% at concentrations below 10 µg/mL. The higher CC_50_ values of AME95 and AME84 compared to α-MG suggest improved safety profiles for these extracts, potentially due to the presence of other bioactive compounds that may modulate cytotoxicity or enhance cellular tolerance.

### 3.2. Antiviral Activity of α-MG and Its Enriched Extracts (AMEs)

The antiviral activity of α-MG and AMEs was screened using non-cytotoxic concentrations (0.5 to 5 μg/mL), as determined from cytotoxicity assays. Compounds were tested at three distinct stages of viral infection: protection (pre-treatment), binding (co-incubation), and post-infection (treatment after infection). The cytopathic effect (CPE) reduction was evaluated using 0.5% crystal violet staining at 48 h (Figure S1). The post-infection assay demonstrated that all the compounds effectively reduced CPE starting from 2 to 5 μg/mL. In contrast, all the compounds showed minimal antiviral activity in the protection and binding assays, suggesting that their primary mode of action occurs after viral entry and during the replication phase (Figure S1). Therefore, α-MG and AMEs were further tested for their efficacy against FIPV infection in the post-infection assay using the immunoperoxidase monolayer assay (IPMA).

### 3.3. α-MG and Its Enriched Extracts (AMEs) Potently Inhibit FIPV Infection in Infected Cells

**Figure 2:**
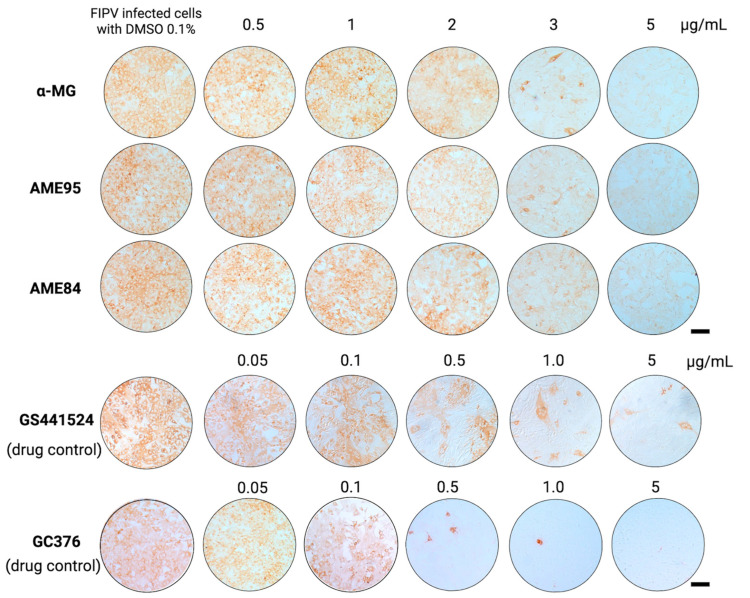
Post-infection antiviral effects evaluated by IPMA with DAB visualization within 24 h. GS-441524 and GC-376 served as positive controls. Scale bars = 200 μm.

To confirm these findings, viral infection was assessed in the FIPV-infected CRFK cells using the immunoperoxidase monolayer assay (IPMA) following the post-infection protocol. Immunostaining revealed a concentration-dependent reduction in cytoplasmic FIPV antigen expression, indicated by decreased brown staining intensity in compound-treated cells (Figure 2). At the highest tested concentration (5 µg/mL), α-MG, AME95, and AME84 all reduced viral antigen expression compared to infected controls. The calculated EC_50_ values demonstrated that all three compounds exhibited potent antiviral activity, which is summarized in Table 2. These values were comparable to those of the positive controls, GS-441524 and GC-376.

### 3.4. Virucidal Effect of α-MG and α-Mangostin-rich Extracts (AMEs)

Based on the initial screening results, we further investigated the direct inactivation potential of AMEs against FIPV. The α-MG and AME compounds were tested at a concentration of 5 µg/mL.

The antiviral activity assay demonstrated that both AME95 and AME84 exhibited significant virucidal activity. AME95 and AME84 reduced the viral titers from 4.67 log TCID_50_/mL to 3.5 log TCID_50_/mL, representing a reduction of 1.17 log TCID_50_/mL for both extracts. In contrast, α-MG demonstrated a minor virucidal effect, reducing the viral titer to 4.5 log TCID_50_/mL (Table 2). These findings suggest that the α-MG-rich extracts, AME95 and AME84, possess direct virucidal properties against FIPV that are more potent than those observed with the α-MG compound. This differential activity implies that other components present in the AMEs may contribute to or enhance the virucidal effect.

To confirm the specificity of antiviral activity, other mangostin derivatives (β-MG and γ-MG) were also evaluated against FIPV. Neither β-MG nor γ-MG exhibited substantial inhibitory activity against FIPV replication at concentrations up to 10 μg/mL (Figure S2), confirming that the antiviral activity is specific to the α-mangostin isomer.

### 3.5. α-MG and α-Mangostin-Rich Extracts (AMEs) Reduced FIPV Replication and Modulated Cytokine Expression in a Dose-Dependent Manner

**Figure 3:**
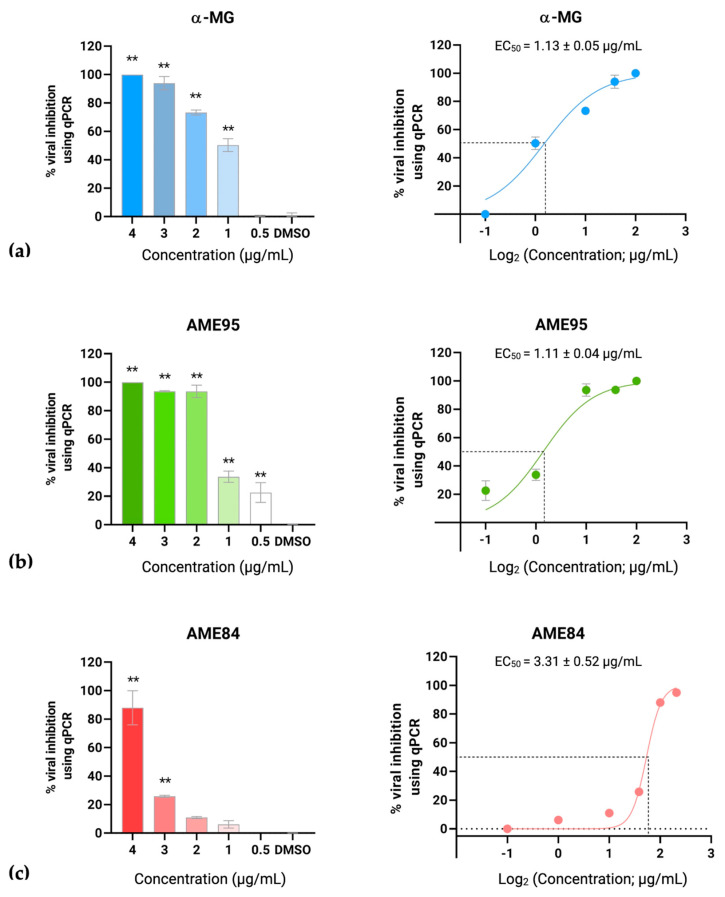
RT-qPCR analysis of FIPV viral load following treatment with (**a**) α-MG, (**b**) AME95, and (**c**) AME84. Left panels show dose-dependent viral load reduction with statistical significance (** *p* < 0.01). Right panels show dose–response curves and EC_50_ values.

α-MG and AMEs exhibited dose-dependent inhibitory effects against FIPV replication. As determined by viral load quantification, α-MG demonstrated potent antiviral activity with 93.99% and 99.99% reductions in viral loads at concentrations of 3 and 4 μg/mL, respectively (Figure 3a). Treatment with AME95 significantly reduced viral nucleic acids by 93.57%, 93.64%, and 99.99% at concentrations of 2–4 μg/mL, respectively (Figure 3b). Similarly, AME84 demonstrated antiviral activity with 25.81% and 88.01% viral reductions at concentrations of 3 and 4 μg/mL, respectively (Figure 3c). The EC_50_ values of the AME compounds were comparable, with α-MG at 1.13 ± 0.05 μg/mL, AME95 at 1.11 ± 0.04 μg/mL, and AME84 at 3.31 ± 0.52 μg/mL (Figure 3a–c).

**Figure 4:**
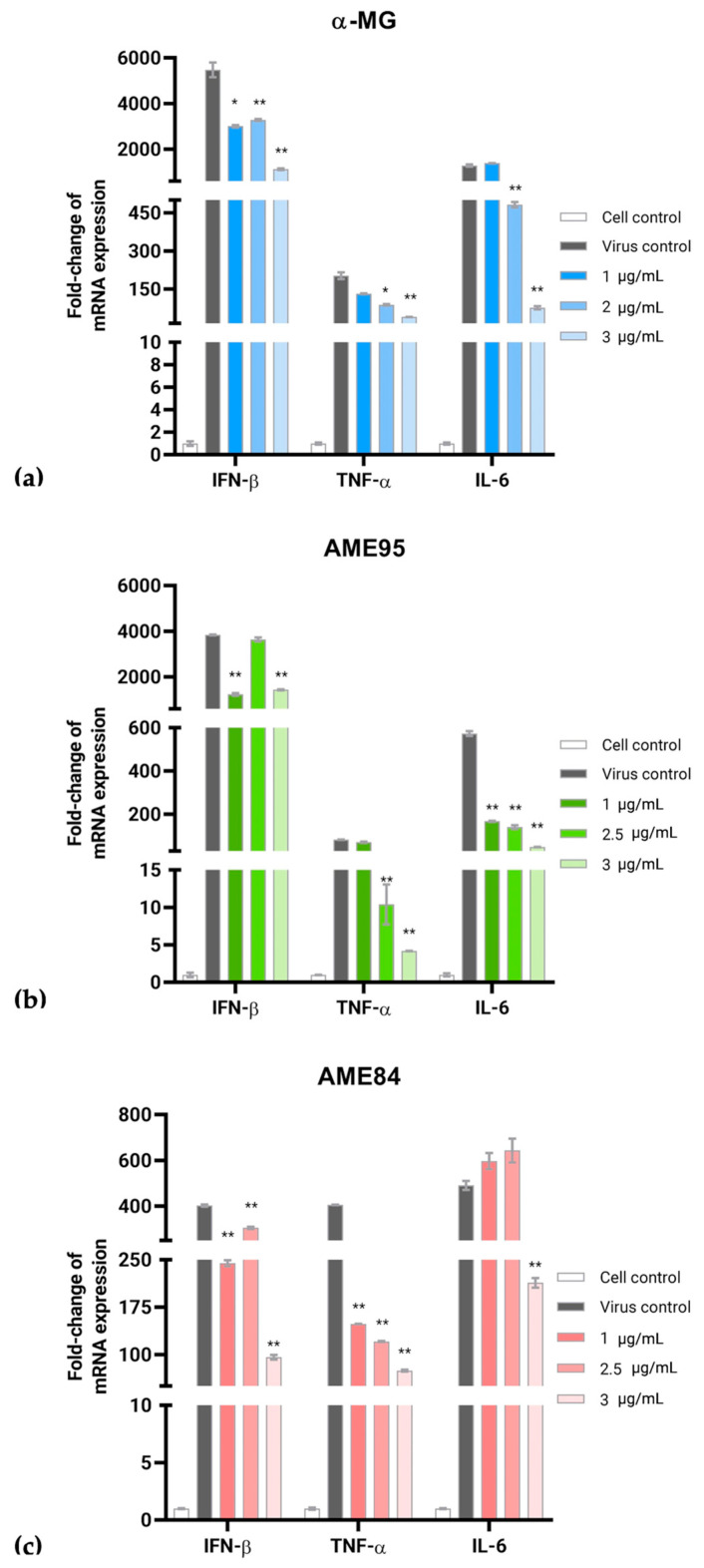
Cytokine expression in FIPV-infected CRFK cells treated with (**a**) α-MG, (**b**) AME95, and (**c**) AME84. Fold-change in inflammatory cytokine mRNA levels analyzed by the ΔΔCt method, normalized to non-infected controls. Statistical significance: * *p* < 0.05, ** *p* < 0.01.

Both α-MG and AMEs significantly modulated the expression of inflammatory cytokines in the FIPV-infected CRFK cells. Following FIPV infection, the expression of IFN-β, TNF-α, and IL-6 was significantly modulated by these compounds in a dose-dependent manner (Figure 4). In the FIPV-infected CRFK cells, α-MG (1–3 μg/mL) significantly altered cytokine expression patterns. Particularly, α-MG at 3 μg/mL resulted in a 4.85-fold reduction in IFN-β mRNA expression, while TNF-α showed a 4.58-fold decrease. The IL-6 mRNA levels were reduced by 16.74-fold compared to the infected controls (Figure 4a). Similarly, AMEs reduced mRNA expression of cytokines, with the AME95 treatment at 3 μg/mL significantly reducing IFN-β mRNA expression by 2.67-fold compared to infected controls. The expression of TNF-α was down-regulated by 19.79-fold, while IL-6 mRNA levels decreased by 11.71-fold relative to infected controls (Figure 4b). Although the AME84 treatment initially showed a slight increase in IFN-β and IL-6 mRNA expression at 1 and 2.5 μg/mL, AME84 at 3 μg/mL significantly reduced the expression of all three cytokines by 4.21-fold for IFN-β, 4.58-fold for TNF-α, and 16.74-fold for IL-6, respectively (Figure 4c). These findings suggest that α-MG and AMEs possess anti-inflammatory properties in addition to their antiviral activities, potentially contributing to their therapeutic efficacy against FIPV infection.

### 3.6. Drug Combination Analysis

The interactions between α-MG, AME95, and AME84 with GS-441524 and GC-376 were evaluated using a comprehensive 5 × 5 dose–response matrix. Cell viability was assessed through complementary methods, combining the CCK-8 assay for precise quantitative measurements and crystal violet staining for qualitative assessment. The CCK-8 results showed high correlation with the crystal violet staining patterns, validating the reliability of the findings.

**Figure 5:**
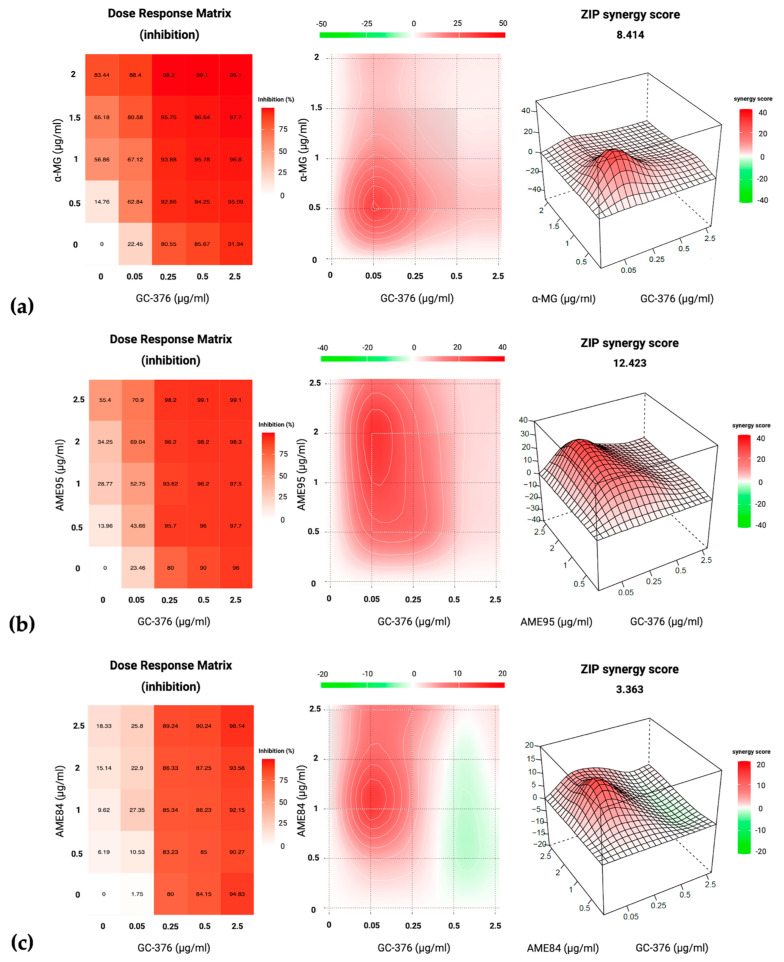
Drug combination analysis using the ZIP model. Synergy scores for interactions between test compounds and GC-376: (**a**) α-MG, (**b**) AME95, (**c**) AME84. Color scale represents % inhibition and synergy scores (red = synergistic, green = antagonistic, and white = additive).

Analysis using the ZIP model in SynergyFinder version 3.0 revealed positive interactions between the tested compounds. The combination of AME95 with GC-376 demonstrated a clear synergistic interaction, with a synergy score of 12.423, indicating strong synergistic effects. This combination enhanced antiviral effects at lower concentrations compared to monotherapies. The most pronounced synergy was observed when AME95 was applied at 0.5–2.0 μg/mL, with GC-376 fixed at 0.05 μg/mL. Similarly, α-MG combined with GC-376 yielded a synergy score of 8.414, indicating a moderate synergistic effect. The optimal activity for this pair occurred at 0.5 μg/mL of α-MG with 0.05 μg/mL of GC-376. In contrast, the combination of AME84 and GC-376 resulted in a synergy score of 3.363, consistent with an additive effect (Figure 5).

Combinations involving GS-441524 generally showed lower synergy scores, reflecting mostly additive interactions. The AME84 and GS-441524 combination exhibited the highest additive score of 7.164 among the GS-441524 combinations tested (Figure S3). Multiple synergistic models were applied to validate these interactions, with consistent results across different analytical approaches confirming the reliability of the observed drug interactions (Table S1).

## 4. Discussion

In this study, we demonstrated the potent antiviral activity of AMEs and α-MG against FIPV. Both AME95 and AME84 exhibited significant antiviral effects with EC_50_ values ranging from 2.80 to 2.88 µg/mL, showing comparable antiviral efficacy to the established control drugs GS-441524 and GC-376. Particularly, the higher purity extract AME95 demonstrated superior antiviral activity compared to AME84, correlating with the higher α-MG concentration.

These findings are consistent with previous studies demonstrating the antiviral properties of α-MG against various viruses, including dengue virus, chikungunya virus, and hepatitis C virus [17,18,19,34,35]. More recently, α-MG was shown to inhibit SARS-CoV-2, another coronavirus, with promising antiviral activity [20]. This broad-spectrum activity against different virus families suggests potential therapeutic applications across multiple viral infections in veterinary medicine.

Our results revealed that α-MG exhibited selective antiviral activity against FIPV, whereas the structurally related compounds β-MG and γ-MG showed negligible effects. This selectivity confirms that the antiviral activity is specific to the α-MG compound, making it the key therapeutic target for extract standardization and quality control in potential veterinary applications.

FIPV infection induces pathogenicity through viral mutations and an excessive immune response, leading to a highly fatal systemic immune-mediated disease [3,4,5]. The fatal outcome of FIP is characterized by elevated inflammatory cytokine expression, with clinical manifestations strongly associated with inflammatory responses [5,9,31]. Studies have demonstrated significant correlations between clinical FIP cases and the expression of various cytokines, including IL-1β, IL-6, IL-10, IL-12, and TNF-α [5,29,36].

Our study demonstrated significant immunomodulatory effects through the suppression of key inflammatory cytokines, particularly IFN-β, TNF-α, and IL-6, in FIPV-infected cells. This dual antiviral and anti-inflammatory activity is particularly relevant to FIP treatment, as the disease involves both active viral replication and destructive immune responses. While cytokine inhibitors such as pentoxifylline and non-steroidal anti-inflammatory drugs have shown promise in reducing vasculitis severity through TNF-α suppression [37], and feline TNF-α-neutralizing monoclonal antibodies have been investigated [38], these immune-suppressing approaches alone have not proven effective in treating FIP. The dual antiviral–immunomodulatory properties of α-MG may address the complex pathophysiology of FIP more effectively than single-target therapies.

Beyond the biological effects, this research also highlights the advantages of environmentally friendly microwave-assisted extraction for producing AMEs suitable for veterinary applications. This green extraction approach reduces the use of harmful organic solvents while ensuring minimal toxic residual solvents in the final product, enhancing its safety profile for animal patients. The method demonstrates improved efficiency, reduced production costs, and enhanced yield of active ingredients compared to conventional extraction methods [11,22,23,39,40,41]. This sustainable production approach aligns with current trends toward safer, more environmentally conscious drug development in veterinary medicine.

Combination therapy using α-MG and AMEs with established antiviral agents like GS-441524 or GC-376 represents a promising strategy for improving FIP treatment outcomes. The predominant additive to synergistic effects observed in our study suggests that such combinations could allow for reduced dosages of individual components, potentially minimizing the dose-dependent nephrotoxicity associated with prolonged GS-441524 treatment while maintaining therapeutic efficacy [1,2,8,9,42]. The combination approach offers several clinical advantages. First, using drugs with different mechanisms of action may reduce the likelihood of viral resistance development, a significant concern in long-term FIP treatment protocols [9]. Second, the dual antiviral and anti-inflammatory properties of α-MG complement the targeted enzymatic inhibition by current FIP therapeutics. Third, this approach may be particularly valuable for treating neurological FIP [7], where achieving therapeutic levels in the central nervous system often requires higher dosages that approach toxicity thresholds [6,7].

From a veterinary practice perspective, combination therapy could potentially reduce treatment duration, improve success rates, and minimize adverse effects—all critical factors in managing this historically fatal feline disease [2,43]. The anti-inflammatory component may also help manage the clinical signs associated with FIP’s immune-mediated pathology.

This study provides compelling in vitro evidence for the antiviral and immunomodulatory properties of α-mangostin and its enriched extracts (AMEs). However, we acknowledge certain limitations of the present study that warrant future research. Firstly, while AMEs were shown to be effective, our study could not fully elucidate the effects of their non-α-mangostin components. Particularly, AME84 contains at least 6.4% (*w*/*w*) of other extractive components, and the differing activities between AME84 and AME95 suggest that these constituents may influence overall efficacy. Secondly, we acknowledge that the antiviral drugs used in combination therapy, such as GS-441524 and GC-376, remain largely unapproved and inaccessible, which presents a practical contradiction to our findings. To address these limitations and advance our promising results toward clinical veterinary application, several comprehensive studies are essential. These include evaluating therapeutic efficacy and safety in naturally infected cats, conducting pharmacokinetic studies to determine appropriate dosing regimens, and developing suitable veterinary formulations for optimal bioavailability. A more practical long-term strategy would be to develop AMEs as single therapeutic agents or explore their combination with other widely accepted supportive care protocols or available conventional drugs. This approach would allow for long-term safety evaluations and integration into current FIP treatment protocols, which will be crucial for successful clinical implementation.

## 5. Conclusions

These findings provide a foundation for developing novel therapeutic approaches that could significantly improve treatment outcomes for this devastating feline disease. The combination of antiviral efficacy, anti-inflammatory properties, and potential for reduced toxicity, coupled with their production through eco-friendly green extraction procedures, makes α-mangostin-rich extracts compelling candidates for further veterinary clinical development.

## Figures and Tables

**Table 2:** The cytotoxicity and antiviral activity assays of α-MG and AMEs.

Compounds	Cytotoxicity (CC_50_; µg/mL) ^1^	Antiviral Activity Assay		
		Post-infection Assay (EC_50_; µg/mL) ^2^	Selective Index (CC_50_/EC_50_)	Virucidal Effect (log TCID_50_/mL) ^3^
α-MG	8.82 ± 0.91	2.71 ± 0.42	3.25	0.17
AME95	10.26 ± 0.11	2.80 ± 0.45	3.66	1.17
AME84	9.85 ± 0.41	2.88 ± 0.46	3.42	1.17
GS-441524 (Drug control)	58.06 ± 0.67	0.62 ± 0.09	93.65	ND
GC-376 (Drug control)	44.47 ± 0.92	0.11 ± 0.03	404.27	ND

^1^ CC_50_ (Cytotoxic Concentration 50) calculated by non-linear regression analysis in a dose-dependent manner, representing the compound concentration that causes 50% cytotoxicity in host cells. ^2^ EC_50_ (Effective Concentration 50) calculated by non-linear regression analysis in a dose-dependent manner, indicating the concentration of the compound that achieves 50% antiviral activity against FIPV in CRFK cells using IPMA. ^3^ virucidal effect calculated by reducing initial viral titer from 4.67 log TCID_50_/mL, with each compound tested at a concentration of 5 μg/mL, demonstrating dose-dependent antiviral potential. ND = Not determined.

## Data Availability

The original contributions presented in this study are included in the article/supplementary material. Further inquiries can be directed to the corresponding author(s).

## Supplementary Materials

The following supporting information can be downloaded at https://www.mdpi.com/article/10.3390/ani15162417/s1. Figure S1. Antiviral activity of α-MG and AMEs against FIPV infection within 48 h: (a) CRFK cells are infected with FIPV and treated with test compounds. (b) Antiviral effects evaluated by protection, binding, and post-infection assays using 0.5% crystal violet staining. Scale bar = 200 μm. Figure S2: Cytopathic effect (CPE) assay of β-MG and γ-MG against FIPV in CRFK cells. (a) Protection assay, (b) binding assay, and (c) post-infection assay. Figure S3: Drug combination analysis of α-MG and AMEs with antiviral GS441524 using the ZIP model. Synergy scores were calculated using SynergyFinder to evaluate interactions between test compounds (α-MG, AME95, and AME84) and established antivirals. Table S1: Drug interaction analysis of α-MG, AME95, and AME84 in combination with established antiviral agents (GS-441524 and GC-376) against FIPV.

## Abbreviations

The following abbreviations are used in this manuscript:

α-MG	α-mangostin
β-MG	β-mangostin
γ-MG	γ-mangostin
AMEs	α-mangostin-rich extracts
CRFK cell	Crandell Rees feline kidney cell
FCoV	Feline coronavirus
FECV	Feline enteric coronavirus
FIP	Feline infectious peritonitis
IPMA	Immunoperoxidase monolayer assay
RT-qPCR	Reverse Transcription quantitative Polymerase Chain Reaction
TCID_50_	Tissue culture infectious dose
ZIP	Zero interaction potency
