# Supplementary material for: Antiviral and Immunomodulatory Effects of α-Mangostin Against Feline Infectious Peritonitis Virus: In Vitro Assay
Source: Animals (Basel). 2025 Aug 18;15(16):2417. doi: 10.3390/ani15162417 (PMC12383049; doi:10.3390/ani15162417)
Supplement: Supplementary file 1 [file animals-15-02417-s001.zip › animals-3781480-supplementary.pdf]

Supplementary Information

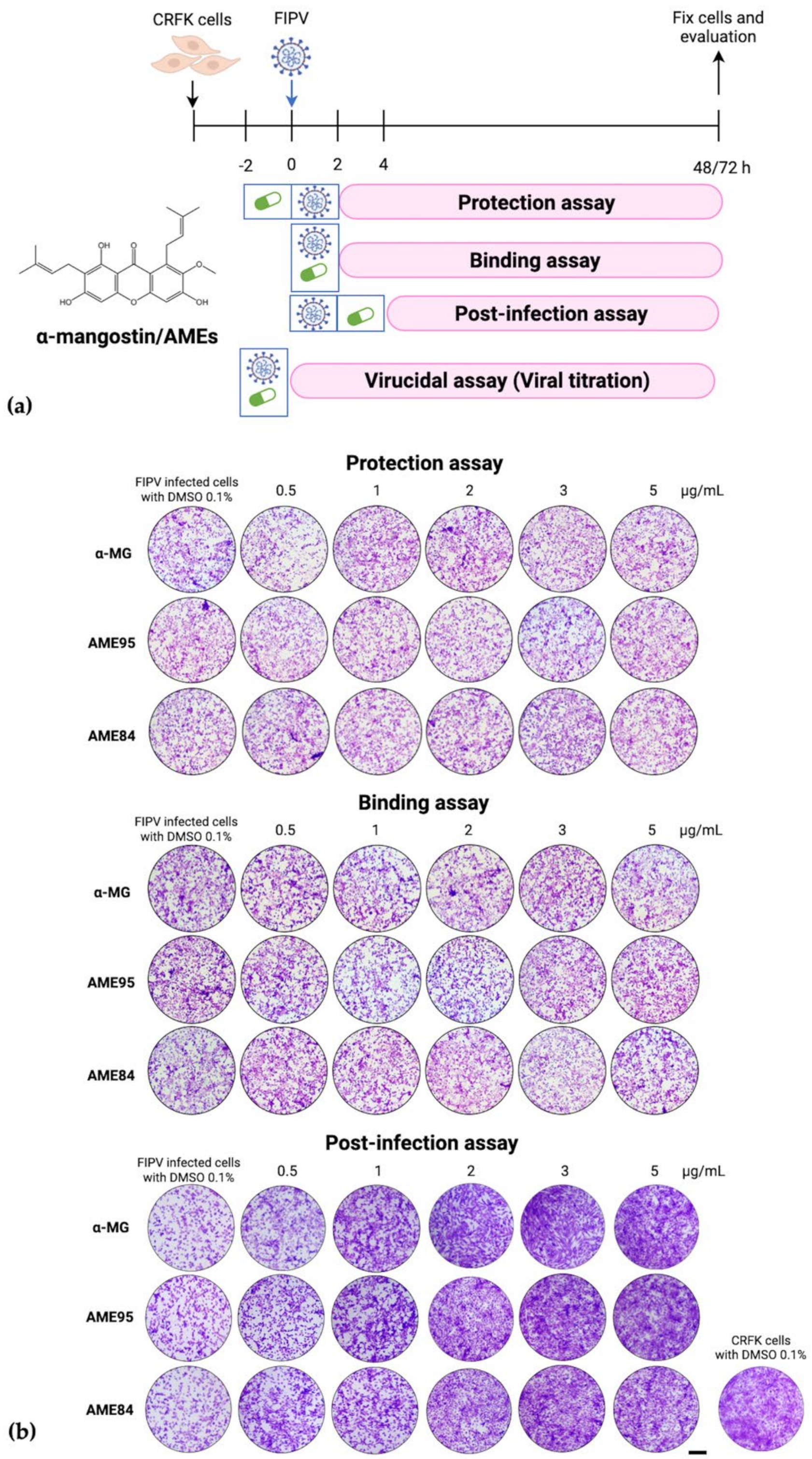

Figure S1. Antiviral activity of *α*-MG and AMEs against FIPV infection within 48 h: (a) CRFK cells are infected with FIPV and treated with test compounds. (b) Antiviral effects evaluated by protection, binding, and post-infection assays using 0.5% crystal violet staining. Scale bar = 200 μm.

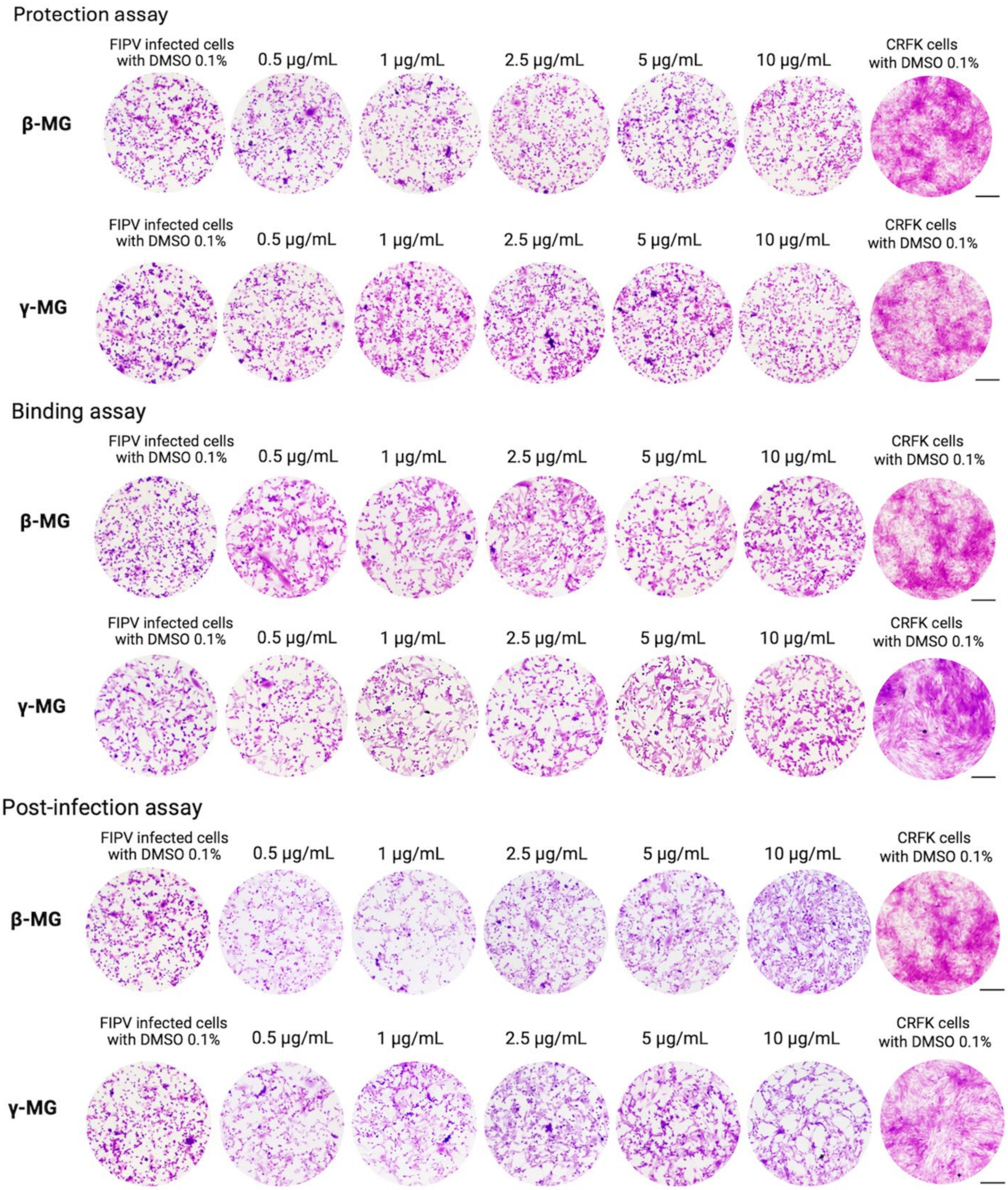

Figure S2. Cytopathic effect (CPE) assay of β-MG and γ-MG against FIPV in CRFK cells. **(a)** Protection assay, **(b)** binding assay, and **(c)** post-infection assay. Cells were treated with the compounds and stained with 0.5% crystal violet at 48 h post-infection. No notable protection against FIPV-induced CPE was observed. Scale bar = 200 μm.

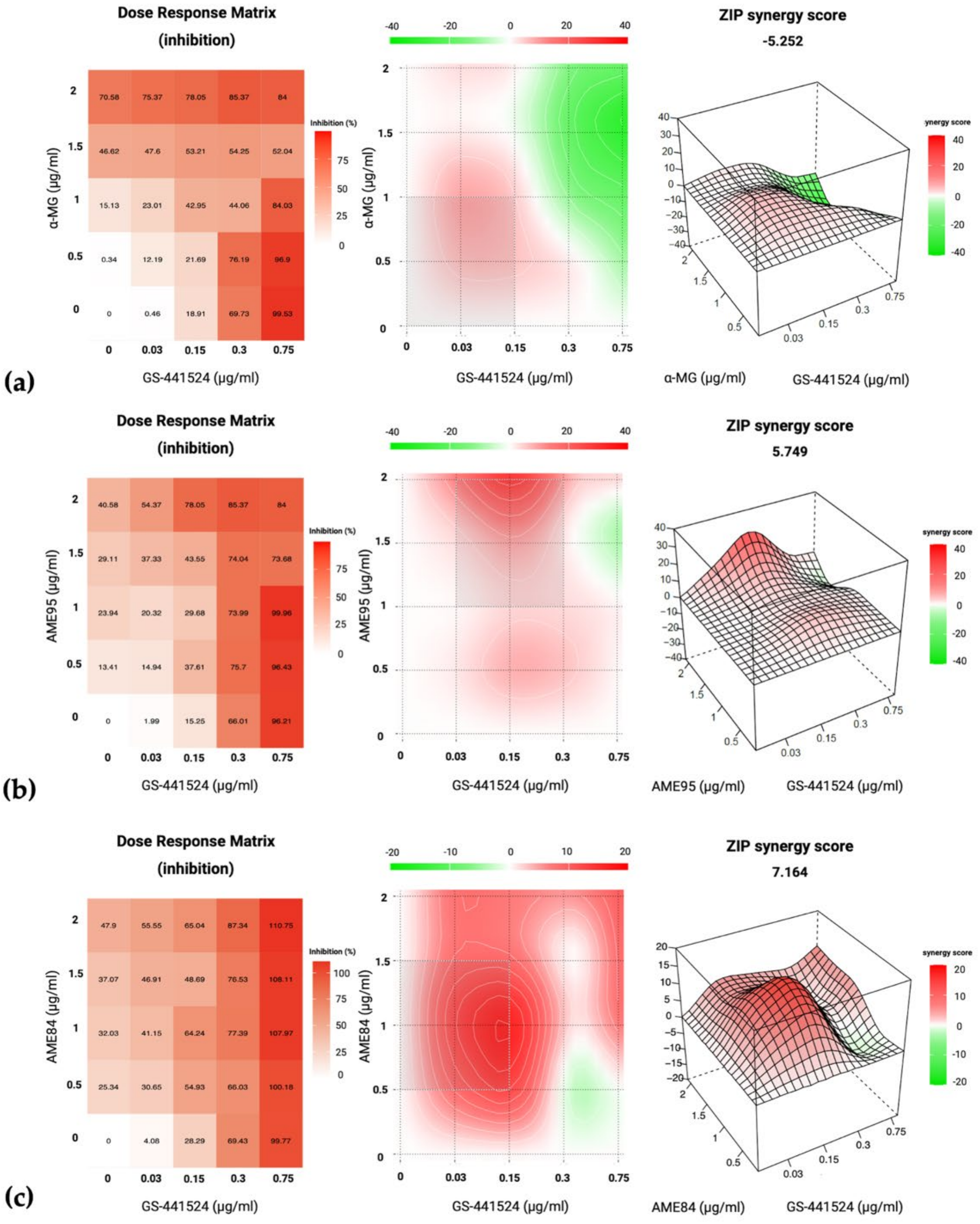

Figure S3. Drug combination analysis of α-MG and AMEs with antiviral GS441524 using the ZIP model. Synergy scores were calculated using SynergyFinder to evaluate interactions between test compounds (α-MG, AME95, and AME84) and established antivirals. Combinations with GS441524: **(a)** α-MG, **(b)** AME95, and **(c)** AME84. The color scale represents % inhibition and synergy scores, where red indicates synergistic effects, green indicates antagonistic effects, and white indicates additive effects.

Table S1. Drug interaction analysis of α-MG, AME95, and AME84 in combination with established antiviral agents (GS-441524 and GC-376) against FIPV. Synergy scoring models including ZIP (Zero Interaction Potency), HSA (Highest Single Agent), Loewe additivity, Bliss independence, and Bliss/Loewe hybrid were used to comprehensively evaluate the combination effects. Values represent interaction scores for each model, with scores >10 indicating synergism, scores between –10 and 10 indicating moderate synergistic or additivity, and scores < –10 indicating antagonism.

Drug combinations	Synergy scoring models				
	ZIP	HSA	Loewe	Bliss	Bliss/Loewe
GC-376 with α-MG	8.414	**14.55**	7.409	8.49	5.482
GC-376 with AME95	**12.423**	**15.598**	–1.749	**12.477**	–2.126
GC-376 with AME84	3.363	4.511	–0.988	3.567	–1.36
GS-441524 with α-MG	–5.252	–1.289	–11.102	–5.760	–13.176
GS-441524 with AME95	5.749	9.107	–0.764	5.285	–1.959
GS-441524 with AME95	7.164	**12.726**	2.152	7.037	1.234

Bold letters: synergism effect
